# Kirsten Ras* oncogene: Significance of its discovery in human cancer research

**DOI:** 10.18632/oncotarget.8773

**Published:** 2016-04-17

**Authors:** Nobuo Tsuchida, Avaniyapuram Kannan Murugan, Michele Grieco

**Affiliations:** ^1^ Graduate School of Medical and Dental Sciences, Tokyo Medical Dental University, Yushima, Bunkyo-ku, Tokyo, Japan; ^2^ Department of Molecular Oncology, King Faisal Specialist Hospital and Research Center, Riyadh, Saudi Arabia; ^3^ DiSTABiF, Dipartimento di Scienze e Tecnologie Ambientali, Biologiche e Farmaceutiche, Seconda Università di Napoli, Caserta, Italy

**Keywords:** Kirsten ras, K-ras, KRAS, human cancer, oncogene, EGFR-targeted therapy

## Abstract

The *KRAS/* K*-RAS* oncogene is crucially involved in human cancer. The term “oncogene” – i.e., a gene able to transform a normal cell into a tumor cell – was introduced in 1969, but the word was not used in the human carcinogenesis literature until much later. Transforming *Kras* and *Hras* oncogenes from the Kirsten and Harvey sarcoma viruses were not identified until the early 1980s due to the complicated structures of the viral genomes. Orthologs of these viral oncogenes were then found in transforming DNA fragments in human cancers in the form of mutated versions of the *HRAS* and *KRAS* proto-oncogenes. Thus, *RAS* genes were the first human oncogenes to be identified. Subsequent studies showed that mutated *KRAS* acted as an *in vivo* oncogenic driver, as indicated by studies of anti-EGFR therapy for metastatic colorectal cancers. This review addresses the historical background and experimental studies that led to the discovery ofKirsten *Ras* as an oncogene, the role of mutated *KRAS* in human carcinogenesis, and recent therapeutic studies of cancer cells with *KRAS* mutations.

## INTRODUCTION

*KRAS*/*K-RAS* is the most frequently mutated transforming oncogene in tumors of the pancreas, and colorectum [1], COSMIC:http://www.sanger.u.k. Indeed, *KRAS* mutations occur in 22% of all tumors analyzed (the highest among *RAS* isoforms), while *HRAS* and *NRAS* mutations are less frequent (3% and 8%, respectively) [2]. *KRAS* was originally identified in Kirsten sarcoma virus (Ki-SV) DNA [3, 4]. It was named *kis*, although the viral *Kras* (v-*Kras*) was first named Kirsten *ras* [5]; its product was identified as a 21 kDa protein (p21) with guanine nucleotide-binding activity [6, 7] in Ki-SV-transformed cells. The protein shared antigenicity with the viral p21 *Hras* oncogene (v-*Hras*), a product of the Harvey sarcoma virus (Ha-SV) [6].

Harvey and Kirsten sarcoma viruses were isolated as sarcoma-inducing retroviruses during the passaging of murine leukemia virus (MLV) in rats [8, 9]. The transforming genes were thought to be derived from the rat genome, while a temperature-sensitive (*ts*) Ki-SV was reported for transformation [10]. The viral RNA sequences were shown to share homology with rat leukemia virus (RaLV) RNA [11, 12]. Subsequently, a rat genome retrovirus-like RNA sequence (VL30) was found to be incorporated into RaLV particles [13, 14]. However, there was no evidence of either gene amplification or the presence of additional sequences in VL30 from rat tumor DNA [14]. Thus, viral *ras* oncogenes could not be identified until these viral genomic DNAs [3, 15] were cloned and sequenced in 1981-1982 [4, 16, 17]. During the same years, the mouse ortholog (*bas*) of v-*Hras* was identified in BALB-murine sarcoma virus (MSV) (BALB-MSV), which had been isolated from a BALB/c mouse hemangio-sarcoma [18, 19].

Meanwhile, studies of human transforming genes were initiated using an entirely different method: DNA transfection. In 1972, transforming activity was reported in cellular DNA fragments transferred into other cells. The DNA had been extracted from hamster cells transformed by the *ts* mutant of Rous sarcoma virus (RSV) [20]. This method was successfully applied in Weinberg's laboratory [21], followed by Cooper's, Wigler's, and Barbacid's laboratories, for mouse and human cancer cell DNA fragments that transformed “normal” mouse NIH3T3 [22–25]

In 1982-3, orthologs of viral *ras* oncogenes with point mutations were identified in transforming DNA fragments from human cancer cells both for *HRAS* [26–31] and *KRAS* [32–34]. This identification of *RAS* genes as oncogenes marked the beginning of molecular oncology in human cancer research. The *Ras* oncogene research was reviewed by Malumbres and Barbacid [35], and retroviral oncogenes were reviewed by Vogt [36]. Other oncogenes first identified in retroviruses and later as drivers in human cancer include *myc*, *raf, erbB1* (*EGFR:* Epidermal growth factor receptor), *AKT,* and *sis* (*PDGF:* platelet derived growth factor, subunit B). Subsequently, these genes were found to be involved in the growth signaling cascade [35].

This review describes (i) the historical background and experimental basis of the “oncogene” concept, (ii) the details of the discovery of the transforming viral and human *KRas* oncogenes along with *HRas/Bas*, and (iii) how the word “oncogene” was integrated into human cancer research literature as one of the most important keywords, according to PubMed database records (http://www.ncbi.nih.gov). Recent advancements in the field and studies of the clinical relevance of *KRAS* as an oncogenic driver in human cancer pathogenesis, and as a therapeutic target, are reviewed.

## TIMELINE VIEW: THE “ONCOGENE THEORY, ” THE DISCOVERY OF VIRAL AND HUMAN *RAS* ONCOGENES, AND THE CLINICAL RELEVANCE OF *KRAS* MUTATIONS

**Figure 1:**
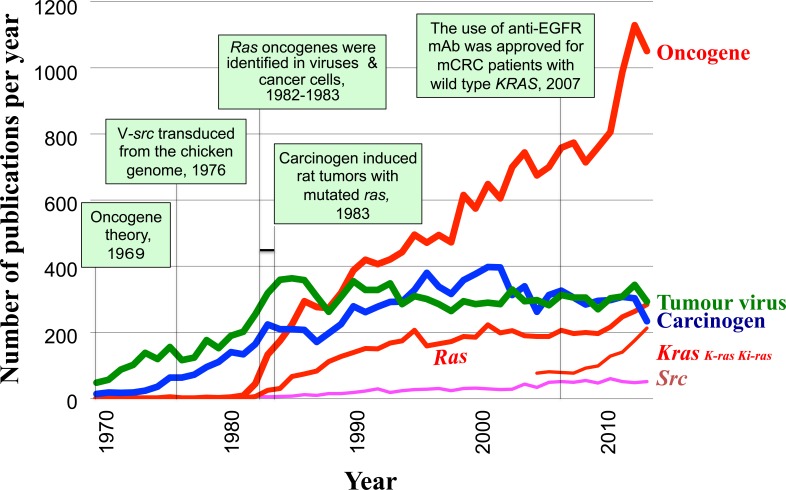
Time line showing the influence of “ras” discovery in human cancer research and the key events related to oncogene history (boxed) Yearly tabulated numbers of publications between 1969 and 2014 with the keywords “human + carcinogenesis” and one of the following: “oncogene, ” “tumor virus, ” “carcinogen, ” “ras, ” “Kras/K-ras/ki-ras, ” or “src.” The numbers for Kras/K-ras/ki-ras were counted separately for Kras, K-ras, or ki-ras, but overlapping publications were only counted once. Numbers were tabulated from 2005, when the Human Genome Nomenclature Committee updated the *KRAS2* (c-ki-*ras*2) gene symbol to *KRAS.* Numbers were counted based on the PubMed database (NCBI, NIH) in July 2015.

Figure 1 shows yearly tabulated numbers of publications extracted from the PubMed database from 1969 to the present, using the keywords “human carcinogenesis” plus one of the following: “oncogene, ” “carcinogen, ” “tumor virus, ” “ras, ” “src, ” or “kras/k-ras/ki-ras.” In 1969, the word “oncogene” was introduced by Huebner and Todaro [37] to explain the mechanism underlying carcinogenesis. The figure shows how the word was integrated into human cancer research.

The first retroviral oncogene (*src*) sequence was found to be transduced from the chicken genome in 1976. The sequence was also present in genomes of other vertebrates, including humans [38], indicating that the *src* retroviral oncogene had a cellular origin. However, Figure 1 shows that “oncogene” or “src” was rarely used as a keyword in human carcinogenesis research literature prior to 1981. Further, the number of publications with “oncogene” began to rise when alterations in cellular oncogenes related to retroviruses were found in human cancer. Transforming *Ras* oncogenes were discovered in the genomes of both Harvey and Kirsten sarcoma viruses and human cancer cells in 1982-1983. Publications using the keyword “ras” were the most common among those using “oncogene.” The discovery of (i) the enhanced expression of the human cellular *MYC* gene (c-*MYC*) following its translocation to the immunoglobulin heavy chain enhancer locus in Burkitt's lymphoma [39], and (ii) the immortalization of primary rat embryo cells by promoter-linked human c-*MYC* [40] also contributed to this rise in “oncogene” studies, as did studies of other retroviral oncogenes [36] and oncogenes in DNA tumor viruses.

At the end of 1983, a chemical carcinogen was found to induce a *ras*-activating mutation in rat mammary tumors [41]. In 1985, the number of publications with “oncogene” as a keyword exceeded those with “carcinogen.” Thus, the word “oncogene” was integrated into human cancer research as one of the most important keywords. During the following two decades, studies of *Ras* were focused mainly on its biological and biochemical characteristics in cancer and normal cells [35]. Additionally, the growth signaling cascade in which Ras is involved was established. This knowledge was later translated into cancer therapies, such as blocking growth factor receptor function [42]. In 2007, the European Medicines Evaluation Agency (EMEA) approved the use of a drug (panitumumab) for anti-EGFR monoclonal antibody therapy for metastatic colorectal cancer (mCRC) patients with wild-type, but not mutant, *KRAS*; *KRAS* mutations were reported to reduce the efficacy of this therapy, as reviewed by Normanno et al. [43]. These findings stimulated research on *KRAS/K-RAS* as shown in Figure 1.

### “Oncogene theory”

### The historical finding as the backbone of the theory

The “oncogene theory” was essentially based on the two main mechanisms of carcinogenesis: chemical and radiation carcinogenesis, and viral carcinogenesis. These mechanisms were discovered in the 1910s, when RSV and coal tar (which contained an “oncogene” and “carcinogens, ” respectively) were shown to induce cancers in experimental animals.

The first keystone discovery for viral carcinogenesis was made in 1911 by Peyton Rous (Rockefeller University). He succeeded in inducing a sarcoma by inoculating RSV in the form of a filtrate of sarcoma tissue extracts into a healthy chicken [44]. Later, Rubin established a method for quantifying transforming activities *in vitro* called a focus assay. The assay was based on the number of transformed cell colonies (focus forming units, FFU) that grew on a flat cell layer of chick fibroblasts [45]. The mouse mammary tumor virus and murine leukemia virus (MLV) retroviruses were also isolated in 1936 and in 1951, respectively. However, an appropriate method to assay the transforming activities of these viruses was not available *in vitro.* The first human cancer virus, Epstein-Barr virus (EBV), was discovered by Epstein, Henle, Achong, and Barr in 1965 [46]. EBV, one of the DNA tumor viruses, induces Burkitt's lymphoma, a cancer of B cells. EBV is involved in lymphocyte immortalization caused by telomerase dysfunction, which is associated with *MYC* activation [47]. Human adenovirus type 12 (Ad 12) was found to induce tumors not in humans but in hamsters, which the virus could infect non-productively. These studies contributed to the elucidation of the general mechanisms underlying cellular transformation. For a review of the history of tumor virology, as reviewed by Javier and Butel [48].

The second keystone discovery for chemical carcinogenesis was made by Yamagiwa and Ichikawa of the University of Tokyo [49]. They succeeded in inducing carcinoma on the ears of rabbits by repeatedly rubbing coal tar, a mixture of many chemical substances, on them. This finding was followed by demonstrations that single compounds in this mixture, such as 3, 4-benzopyrene and α-aminoazotoluene, induced tumors in experimental animals [50, 51]. Chemical carcinogens exhibited genotoxic activities, leading to DNA lesions. Loeb and Harris reviewed advances in chemical carcinogenesis [52].

Extensive overlap was noticed between carcinogens and mutagens [53]. Although these studies did not clearly indicate the existence of a gene(s) that might be directly involved in carcinogenesis, studies from Weinberg's laboratory did [21]. DNA extracted from mouse cancer cells, which had been transformed with chemical carcinogens, were found to induce “foci” upon transfection into “normal” NIH3T3 mouse cells. This observation suggested the presence of a single DNA fragment with transforming activities. This finding was consistent with the idea that a gene(s) was mutated by the carcinogen, thereby conferring transforming activities on a single fragment of the mouse cell DNA.

#### Experimental basis for the theory

As described above, Huebner and Todaro (National Cancer Institute, NIH) proposed the “oncogene theory” in 1969, in a paper titled “Oncogenes of RNA tumor viruses as determinants of cancer” (RNA tumor viruses were later named retroviruses) [37]. It had been proposed that carcinogens could solely explain the development of human cancers, as well as the increased incidence of cancer associated with aging [54].

“Oncogene theory” was based on cancer (mostly leukemia) incidence in the following different inbred mouse strains: AKR (high incidence), BALB/c (intermediate incidence), and C57BL (low incidence). Without exposure to other known cancer risk factors, the incidence was strain-specific and depended on age. Furthermore, incidence levels paralleled the expression levels of endogenous retrovirus and the viral antigen. In addition, type C viruses, similar to RSV particles, were found in human and other vertebrate cells. The presence of retroviral information (endogenous virus) in vertebrate genomes was hypothesized based on these findings. The abstract of this study states: “It is postulated that the viral information (the virogene), including that portion responsible for transforming a normal cell into a tumor cell (the oncogene), is transmitted from animal to progeny animal and from cell to progeny cell in a covert form. Carcinogens, irradiation, and the normal aging process all favor the partial or complete activation of these genes.”

By postulating the presence of oncogenes in the cellular genome, this theory offered an explanation of the mechanisms of carcinogenesis induced by carcinogens and by endogenous viral oncogene genetic sequences, along with aging. The theory was also supported by knowledge of the RSV genome, as investigations of both the “virogene” and the “oncogene” had been reported by that time. Although RSV is an exogenous virus, RSV genetic information could exist in host genome as a provirus [55] that remained after viral infection.

Two criticisms of the “oncogene theory” surfaced later: (i) the relationship between the production of endogenous viruses and cancer incidence was clearly shown only in mouse models, and (ii) the oncogene (*src*) and “virogenes” were found on different chromosomes in chickens [56]. Therefore, the term “oncogene” was rarely used to refer to a transforming gene, while “*src*” was used as a synonym for “transforming gene (oncogene).” However, in 1981, the Retrovirus Study Group of the International Committee on Taxonomy of Viruses named at least 13 distinct *“src”* genes as individual oncogenes, to avoid confusion [5].

### Road to viral *Kras*/*K-ras* oncogene identification

#### Transforming virus-specific sequences of rat, mouse and avian sarcoma viruses studied in the 1970s

One year after the “oncogene theory” was proposed, reverse transcriptase was discovered in retroviral particles [57, 58]. This discovery not only supported the provirus hypothesis, but also made it possible to study the origins of transforming (oncogene) sequences in sarcoma viruses to test the validity of the “oncogene theory”. The replication mechanism of leukemia viruses, which had not yet been clarified, was also studied. Thus, cDNA prepared from viral genomic RNA was used to identify viral oncogene sequences in sarcoma viruses, as well as sequences needed for viral replication (*gag, pol,* and *env*).

**Figure 2:**
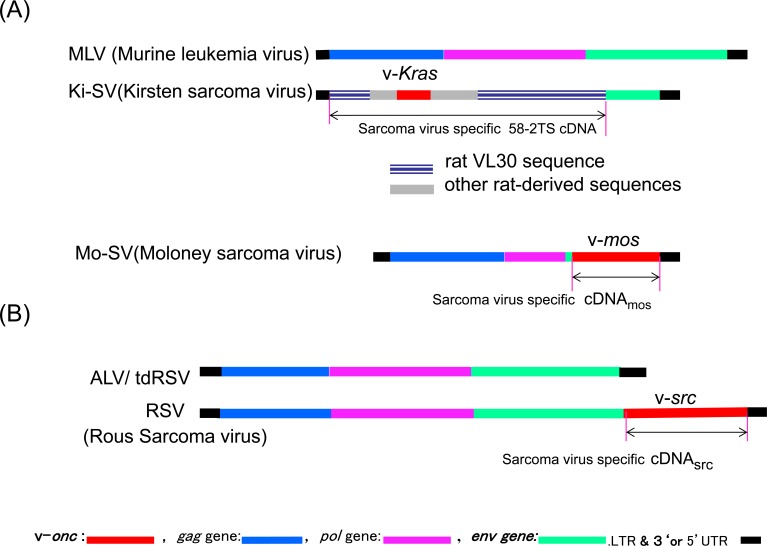
Comparisons of Ki-SV, Mo-SV, RSV, and the corresponding leukemia viral genomes **A.** Genomic structures of Murine leukemia virus (top structure), Kirsten sarcoma virus [17, 72], and Moloney sarcoma virus [141]. **B.** ALV/tdRSV (avian leukemia virus/transformation defective RSV, the upper structure) and RSV (bottom structure). Leukemia viruses/tdRSV mutant genomes are shown for comparison. Each sarcoma virus figure shows regions covered by sarcoma virus-specific cDNA by an arrow.

Sarcoma-virus specific sequences were first prepared from viral particles produced by mouse 58-2T cells, developed by Masakazu Hatanaka, which produced a large excess of Ki-SV over helper MLV [59, 60]. As described earlier, Ki-SV production might have resulted when MLV transduced a sarcomagenic rat sequence (see Figure 2A). Ki-SV specific 58-2TS cDNA was prepared from 58-2T viral cDNA by removing the hybridizable MLV sequence with hydroxyapatite [59]. 58-2TS cDNA detected RNA species that were the same size as 30S RNA (this RNA species was later named VL30) in normal rat cells and RaLV-producing cells [13], consistent with the idea that the Ki-SV was formed by transducing the VL30 sequence. However, most of the 58-2TS cDNA sequences were later found to be those of rat VL30, while less than one-fifth were estimated to be the v-*Kras* oncogene sequence [17] (Figure 2A). It was thus difficult to identify the cellular *Kras* sequence in normal rat cells at that time. However, this strategy of preparing sarcoma-virus specific cDNA was also used to identify the RSV and Mo-SV oncogene sequences as described below.

Two years later, Stehelin, Varmus, Bishop, and Vogt [38] reported the isolation of RSV-specific cDNA (cDNA*_src_*). As shown in Figure 2B, the RSV genome contained the transforming sarcoma virus-specific sequence (*src*) in addition to *gag, pol,* and *env*, which make RSV replication-competent. cDNA fragments complementary to *gag, pol,* and *env* sequences were removed from RSV cDNA preparation. The resulting cDNA*_src_* formed a duplex with chicken genomic DNA at the highest melting temperature (Tm). The duplex with other vertebrate DNAs, including human DNA, had a lower Tm relative to evolutionary distance to the chicken, indicating that the *src* sequence originated in the chicken genome and was conserved in vertebrates, including humans. However, it was difficult to find biochemical differences in *src* genes between normal and cancer cells at that time. Moreover, the *src* sequence in human cancer cells was not known to have transforming activities.

Although transforming human *src* stop-codon mutations were reported in colon cancer more than 10 years later, additional analyses failed to confirm these and other activating transforming mutations, as reviewed by Russello and Shore [61]. As mentioned earlier, the 1976 finding thus did not lead to an immediate increase in publications with “src” or “oncogene” as a keyword in human carcinogenesis research (see Figure 1). However, accumulating evidence showed that cellular SRC kinase expression levels and/or activity were often enhanced in human cancer. It has been reported that src family kinases phosphorylated tyrosine residues of the Bcr-Abl protein, which conferred oncogenic activities [62].

It should be mentioned that the src protein was the first tyrosine kinase to be discovered, as reviewed by Hunter [63]. This led to the discovery of the src family of receptor tyrosine kinases (RTK), as reviewed by Parsons and Parsons in 2004 [64]. The SH (src homology) domain was later found to be crucial for binding to other cellular proteins involved in signaling, as reviewed by Pawson and Schlessinger [65].

Also in 1976, Frankel, Heubauer, and Fischinger reported the isolation of Mo-SV-specific cDNA (then denoted cDNA_sarc_) [66] (see Figure 2A). At that time, *sarc* was used to denote the cellular counterpart of viral *src* (oncogene). The Mo-SV-specific sequence, later named *mos*, was found to have originated from the mouse genome and to be present in the human genome as well [67]. However, the incidence of *mos* mutations was low in human cancer cells, while enhanced expression was observed [68]. It was reported that mos kinase activated ERK in the Ras-Raf pathway [69]. In addition, the roles of c-*mos* in meiotic maturation were shown, as reviewed by Vande Woude [70].

#### Molecular cloning of genomic DNA of sarcoma viruses carrying v-*Kras* or v-*Hras*

**Figure 3:**
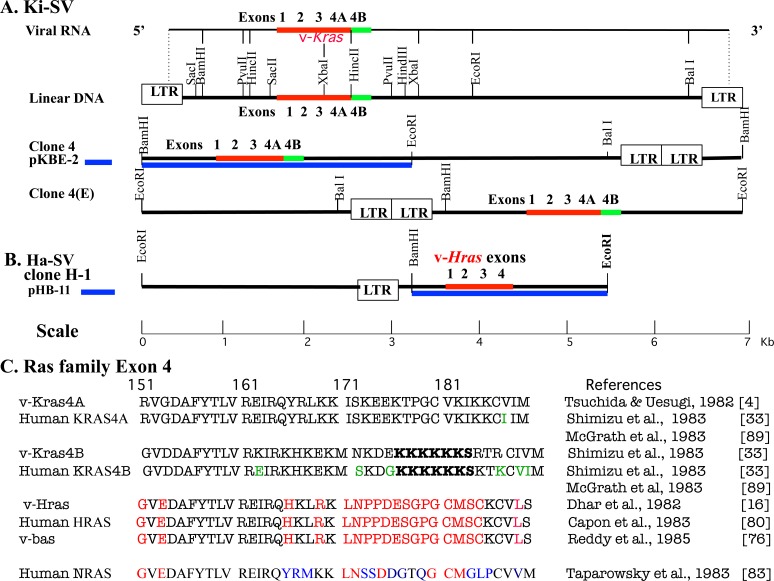
Physical maps and comparison of viral and cellular amino acid sequences of Ras isoforms **A.** Physical map of Ki-SV and its restriction sites [3, 4, 17, 33, 60]. The positions corresponding to *KRAS* exons 1, 2, 3, and 4A, and 4B are shown in red and green, respectively, for (1) viral RNA, (2) the linear form DNA with LTRs at both ends, aligned with viral RNA, (3) clone 4 DNA (circular DNA was linearized at BamHI and inserted into plasmid vector) with subclone KBE-2 underneath as the blue thick line, and (4) clone 4(E) DNA (circular DNA was linearized at EcoRI and inserted into a vector). Restriction sites are shown for viral DNA. **B.** Physical map of Ha-SV DNA Clone H-1 [15, 16, 17, 75, 80]. Circular DNA with LTR was linearized at EcoRI, and the positions corresponding to *HRAS* exon*s* 1, 2, 3, and 4 (in red) and restriction sites are shown. The sub-clone pHB-11 for heteroduplex analysis is shown with the blue thick line, underneath clone H-1. The scale is in Kb for both Ki-SV DNA and Ha-SV DNA. **C.** Comparisons of viral and cellular exon 4 amino acid sequences of Ras isoforms and KRAS exon 4B. Amino acid sequences of v-KRAS, v-HRAS and v-BAS, and those corresponding human sequences of exon 4 and NRAS are presented. Viral KRAS exon 4B is the sequence of the corresponding rat sequence inferred from the corresponding viral sequence [33]. Amino acid residues that are different from that of viral KRAS are shown in red or blue, and differences between the human cellular and the viral sequences are shown in green. In viral and cellular KRAS 4B, polylysine residues and serine 181 phosphorylated with PKC are shown with bold letters.

Molecular cloning of the Ki-SV DNA was conducted in Tsuchida's laboratory at the Wistar Institute in Philadelphia when NIH guidelines for recombinant DNA experiments on retrovirus genomes were relaxed from the P4 to the P2 level (Federal Register Dec 22, 1978 part VI: DHEW NIH, recombinant DNA research, revised guideline). Unintegrated circular genomic DNA of Ki-SV was cloned into a plasmid vector (clone 4) [3, 71]. The linear form of the Ki-SV DNA with restriction sites, aligned with the 5′ to 3′ orientation of viral RNA [60], and both clone 4 and clone 4(E) inserted, as shown in Figure 3A. As a result of the transforming activities of restriction-enzyme-cleaved clone 4(E), at least one XbaI site and one PvuII site were involved in transformation [3]. This result is not consistent with the findings of Chien et al. [72], who reported that *v-Kras* had been estimated to be in the 5′ terminal 0.7 Kb region of viral RNA, since the corresponding region of the viral DNA contained neither a XbaI nor a PvuII site [3] (Figure 3A).

Molecular cloning of the Ha-SV DNA carrying v-*Hras* was conducted in the P4 Mobile Containment Laboratory at the NIH in Bethesda before the NIH guidelines were relaxed. The results were reported by Scolnick and his colleagues [15]. Unintegrated circular genomic Ha-SV DNA was inserted into a lambda phage vector (clone H-1) (Figure 3B). In 1980, the transforming gene of the Ha-SV genome was located in a region between 0.4 kb and 1.5 kb from the 5′ end of the viral RNA genome [73].

A molecular clone of BALB-MSV DNA was also isolated (clone p9) [74]. BALB-MSV DNA had the transforming gene *bas*, the mouse ortholog of v-*Hras* [19], as described above.

#### Comparisons of Ki-SV and Ha-SV and their sub-clones to detect human *KRAS* or *HRAS* sequences

To localize the viral *ras* oncogenic sequences, the cloned Ki-SV and Ha-SV DNA segments (pKBE-2 and pHB-11, respectively), shown as thick blue lines in Figure 3A and 3B, respectively, were compared by heteroduplex analysis, since the ras proteins and genomes of both viruses shared common antigenicity and VL30 sequences, respectively [6, 72]. However, there was a discrepancy between the finding of Chien et al. [72], and that of Tsuchida and Uesugi [3], as described earlier. The analysis showed homology of a short 0.35-kb region, 1.3-1.65 Kb from the 5′ end of Ki-SV RNA, but not the 5′ terminal 0.7 Kb region. The upper XbaI recognition sequence of Ki-SV DNA was placed adjacent to 3′ side of this common region [17] (see Figure 3A). Further, this common region was later found to be highly homologous to the G domain (165 N-terminal amino acids) of v-Kras and v-Hras, as described below. Common tryptic peptides of both Kirsten and Harvey p21 proteins were suggested to be encoded by this region [17]. Consequently, two DNA fragments that encompassed the common region were subcloned from K-SV clone 4: the 1.0 kb HiHi-3 and the 0.38 kb HiHi-380 [17].

**Figure 4:**
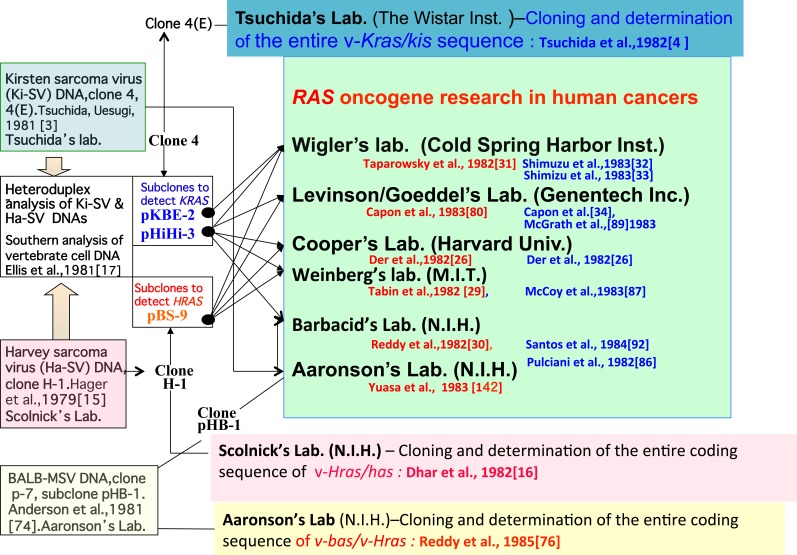
Identification of the viral *ras* oncogenes and distribution of sub-clones Cloning of viral genomes and identification of their oncogenes were performed in the laboratories of Tsuchida (v-*Kras*), Scolnick, (v-*Hras*), and Aaronson (v-*Bas/v-Hras*). Subclones containing viral oncogenes (pHiHi-3, and/or pKBE-2 of v-*Kras*, pBS-9 of v-*Hras)* were used in the laboratories of Wigler, Cooper, Weinberg, and Levinson/Goeddel. The laboratories of Aaronson and Barbacid used their BALB-MSV clone pHB-1 for *HRAS/BAS* detection and clone 4(E) and HiHi-3 for *KRAS* detection. Publications of *RAS* genes from these eight laboratories are listed as references (indicated in red for *HRAS/BAS* and in blue for *KRAS*.

Furthermore, HiHi-380 of v-*Kras* and BS-9 [75] of v*-Hras* (both of these clones lack sequences corresponding to genomic exon 4 sequences) were able to detect respective EcoRI fragments of different sizes in vertebrate genomes; thus, it was possible to distinguish human DNA fragments from mouse DNA fragments in Southern hybridization [17]. HiHi-3 and KBE-2 clones for v-*Kras* and BS-9/pHB-1 for v-Hras/v-bas were used to identify human *KRAS* or *HRAS* exon-containing fragments of NIH3T3 cells transfected with human cancer DNAs (Figure 4). pHB-1 was reported to have sequences to detect all 4 *HRAS* exons of human cancer cells [74, 76].

#### Identification of the oncogenes (v-*Kras*/*kis* and v-*Hras*/*has*) in the Kirsten and Harvey sarcoma viral genomes

Scolnick and his colleagues identified the 1.0 kb nucleotide segment of the Ha-SV DNA encompassing the 0.35 Kb region that duplexed with the Ki-SV DNA. The v-*Hras/has* ORF in this segment was found to encode a 30 kDa protein starting 156 nucleotides upstream of the ATG codon of the internal p21 ORF [16] (Figure 3B). Later, the two upstream initiation codons were shown to be unnecessary for transforming activities [77]. The viral *Kras/kis* gene was localized by analysis of the transforming activities of deletion mutants of clone 4(E). Based on this, the v-*Kras* ORF was localized to the region shown in Figure 3A [4]. This ORF encoded 189 amino acids. Both v*-Hras* and v*-Kras* sequences were presented in the same issue of Science. Portions of the p21 ORF sequences of both v*-Kras* and v*-Hras* were verified in analyses of the tryptic peptides [17, 78].

v-Kras and v-Hras amino acid sequences of p21 ORFs revealed 90% homology in the N-terminal G domain, while the carboxyl terminal 166 to 189 region (highly variable region: HVR) [79] only shared 27% homology (Figure 3C). Therefore, the 0.35 kb homologous region detected by heteroduplex analysis between the two viral DNAs was confirmed to be within the G-domain sequences. When HVR amino acid sequences of the human exon 4 (E4) of HRAS [80] and exon 4A (E4A) of KRAS [33] were compared to the respective viral proteins, they were 100% and 96% similar, respectively, (Figure 3C), confirming that *KRas* and *HRas* were highly phylogenetically conserved independent genes. They were located on different human chromosomes [81, 82].

In 1983, Wigler and his colleagues isolated a new transforming *NRAS* oncogene in a neuroblastoma cell line, and it shared extensive homology with both v*-Kras/KRAS* and v*-Hras/bas/HRAS* [83]. They also identified a common intron-exon structure with 4 exons, except for *KRAS*, which had two 4^th^ exons, 4A and 4B and highly homologous sequences in the G domain. However, the C-terminal HVR sequences were unique for each RAS protein (Figure 3C). The highly homologous G-domain encoded both GTPase [84] and the sequence necessary for binding common downstream effector proteins, as reviewed by McCormick and Wittinghofer [85].

### Road to the first human *RAS* oncogenes

#### *HRAS*/*BAS* oncogene

The following timeline describes the discovery of the ortholog of a retroviral *Hras/bas* oncogene as the first transforming oncogene in human cancer cells.

In 1979, two important discoveries eventually led to the identification of the first human oncogene: (a) the identification of transforming activities in a DNA fragment of carcinogen-transformed mouse cell [21], and (b) molecular cloning of Ha-SV genomic DNA [15]. In 1981-1982, transforming DNA fragments from human cancer cell lines were reported [22–25]. They were molecularly cloned using a human-specific *Alu* sequence as a probe. In 1980 and 1981, viral probes (BS-9 and pHB-1) were constructed from Ha-SV and BALB-MSV DNA, respectively [75, 74].

In 1982, the viral *Hras/bas* probes identified a ~7 kb BamHI fragment in transforming human DNA [26–28]. The V-*Hras* ORF sequence for p21 was reported [16].

A difference was found in a single base of the 12^th^ codon in the 1^st^ exon that conferred transforming activities on the gene. This difference resulted in the substitution of glycine for valine. This was reported by the Weinberg [29], Barbacid [30], and Wigler [31] groups.

It should be noted that the first report of a *HRAS/BAS* codon 12 mutation from Weinberg's group was carried out as collaboration with Scolnick's group [29]. The v-*Hras* BS-9 probe was used in the laboratories of Weinberg, Cooper, Wigler, and Levinson, while viral pHB-1 was used to identify the transforming *BAS/HRAS* fragment in the laboratories of Barbacid and Aaronson (Figure 4).

#### *KRAS*/*K-RAS* oncogene

Structure and activation mechanism of *KRAS* oncogene were elucidated in the following timeline.

1. In 1981 the structure and functions of Ki-SV genomic DNA clones were reported [3], though cloning of Ki-SV DNA corresponding to clone 4 (Figure 3A) was presented at the Cold Spring Harbor meeting in May, 1980 [71].
2. In 1981, the approximate position of v-*Kras* was estimated based on a comparison with Ha-SV genomic DNA. Based on this research, a subclone (HiHi-3) was constructed as a v-*Kras* probe [17] used to detect human *KRAS* DNA in NIH3T3 transfectants of human cancer cell DNAs (see Figure 4).
3. In 1982, Cooper's group [26] detected a transforming human DNA fragment homologous to v-*Kras* in a lung cancer cell line, LX-1. Similarly, Barbacid, Aaronson, and colleagues detected the *Kras* transforming fragment in additional human lung, colon, gall bladder, pancreas, and rhabdomyosarcoma cell lines [86]. The nucleotide sequence of the viral *Kras/kis* ORF was reported [4].
4. In 1983, the Wigler [32] and Weinberg and Lowy [87] groups found a transforming human *KRAS* DNA fragment in Calu-1 and SW480 cancer cells using the v-*Kras* probe and/or the cellular *KRAS* probe (a c-ki-ras2 [88] fragment originally isolated with a v-*Kras* probe), respectively. Wigler's group cloned a transforming *ALU*-containing DNA fragment, in which the v-*Kras* KBE-2 probe mapped the exon-containing EcoRI fragments. An activating point mutation was found in *KRAS* codon 12 in Calu-1 cells [33]. Based on the findings that Calu-1 and SW480 contained a transforming *KRAS* DNA fragment, Levinson's group isolated *KRAS* cDNA clones from both cell lines with a v-*Kras* probe and identified codon 12 mutations with amino acid substitutions by comparing the *KRAS* cDNA sequences in normal and cancer cells [34].

Additionally, Levinson's laboratory constructed a restriction map of the *KRAS* gene in normal human cells [89]. Wigler's and Levinson's groups found that the human *Kras* gene contained exons 1+2+3+4A+4B. The two 4^th^ exons, A and B, resulted in the synthesis of two mRNA species by alternative splicing [33, 89]. Viral pKBE-2 also contained sequences corresponding to exons 1+2+3+4A+4B [33], but the ORF corresponding to exons 1+2+3+4A was possibly translated [4].

Activating mutations in *KRAS*, the transforming fragment of which was 5 times larger, were identified 10 months after the *HRAS* oncogene. However, once the v*-Kras* ORF sequence was published [4], researchers could easily elucidate its structure and point mutations in cancer cells. Viral Kras and the corresponding human protein KRAS^4A^ also had 189 amino acid residues with differences at only 7 positions, one of which was a codon 12 mutation in both genes [4, 33, 89, 90].

#### *Ras* genes in normal cells, cancer cells, and retroviruses

Analyses of human cancer cell *RAS* genes showed that they differed from those in normal cells in single nucleotides of codons, such as 12, 13, 59, 61, and 63; hot spot mutations in exons 1 and 2 [91] were associated with transforming activities. Consequently, transforming cellular genes were also called oncogenes based on the same criteria used to identify viral oncogenes, while *RAS* in normal cells was called a “proto-oncogene.” Thus, the word “oncogene” in cancer cells described a gene that could “transform a normal cell into a tumor cell, ” as first defined by Huebner and Todaro [37]. However, proto-oncogenes and oncogenes were later shown to play unique signaling roles in both normal and cancer cells, respectively, (see below) [35]. In addition, a *KRAS* mutation detected in a lung carcinoma was not present in normal tissues from the same patient, indicating an association between activation of oncogenes and the development of certain human cancers [92]. Additionally, the identification of human transforming genes that corresponded to viral *Hras* and *Kras* suggested that human *RAS* oncogenes could induce cancer just as Ki-SV and Ha-SV did in rodents.

In addition to point mutational activation, three other events are involved in the activation of a proto-oncogene causing enhanced expression of the proto-oncogene product, as reviewed by Vogt [37]: (1) gene amplifications, (2) promoter/enhancer insertion in the vicinity of the proto-oncogene, and (3) chromosomal translocation. Among these, *RAS* gene amplification has been reported in various tumors [93], but events (2) and (3) have not been reported. Chromosomal translocation also results in the formation of an activated oncogene product as a fusion protein, such as *BCR-ABL* [94] and *RET-PTC* [95]. Moreover, leukemia viruses induce transduction either through oncogenes/proto-oncogenes or by generating a gag-fusion gene, thereby changing into transforming viruses. This process was thought to be important for the generation of RSV, other sarcoma viruses, and acute leukemia viruses [37]. The oncogene (*p29 gag-ras*) for Rasheed rat sarcoma virus (RaSV) is reportedly a fusion gene of RaLV *gag* with rat cellular *Hras* [96].

In Ki-SV and Ha-SV, two transduction steps might have occurred: (i) cellular *ras* was inserted into the endogenous rat VL30 retrovirus-like sequence, and (ii) VL30 RNA with *ras* was subsequently transduced into the MLV genome [13, 17, 74 97].

#### Merging of “carcinogenesis” research with oncogene research

As shown in Figure 1, Barbacid and colleagues found, in December 1983, an activated *Hras* oncogene in a mammary carcinoma that was induced in rats with N-nitroso-N-methylurea (NMU), a chemical carcinogen [41]. This finding revealed the following molecular mechanism underlying *in vivo* chemical carcinogenesis: (i) the carcinogen causes mutagenic damage of a proto-oncogene DNA [9], (ii) the proto-oncogene is fixed as an oncogene in the cell genome, and (iii) tumor formation originates from a cell bearing the activated oncogene. Thus, this finding led to the merging of carcinogen research with oncogene research, thereby consolidating human carcinogenesis research and oncogene research at the molecular level (see Figure 1). Consequently, the series of findings on *ras* oncogenes, together with other retroviral oncogenes, greatly influenced human cancer research, and these findings opened up the new research field of molecular oncology.

### Clinical relevance of *KRAS* mutations

#### *KRAS* is the most important biomarker in anti-EGFR therapy for mCRC

**Figure 5:**
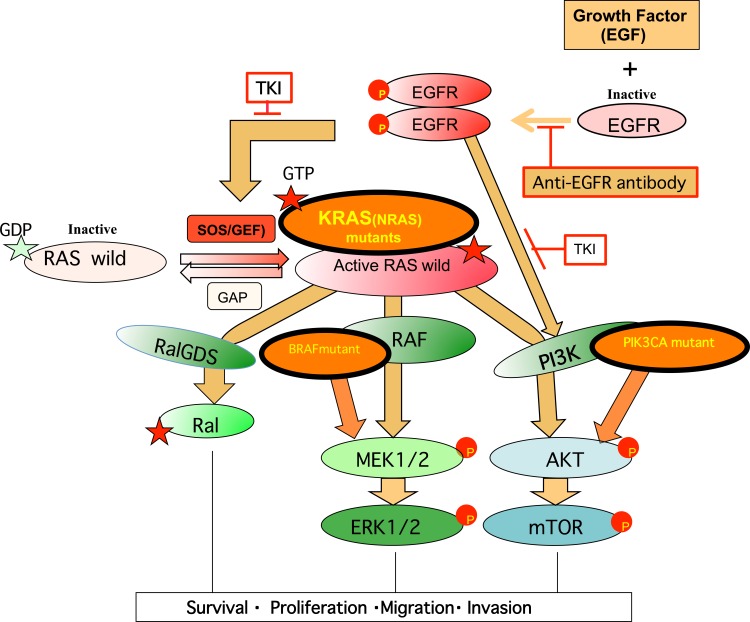
Growth signaling and mutated signal proteins that confer resistance to anti-EGFR-targeted therapy in mCRC Growth factor stimulation in the normal cell generally proceeds as follows: (i) activation of the receptor by phosphorylation and dimerization, (ii) activation of RAS and PI3K, and then (iii) activation of the RAS effector pathways RAF, PI3K, and RALGDS through binding to RBD of each protein. In mCRC, *EGFR* copy number was often increased. EGFR-targeted therapy prevents steps (i) and (ii) by using anti-EGFR monoclonal antibodies (mAb) or a tyrosine kinase inhibitor (TKI). An oncogenic driver mutation of the EGFR effector (*KRAS, NRAS*, or *PIK3CA*) or the RAS effector (*PIK3CA* or *BRAF*) makes cancer patients refractory to EGFR-targeted therapy, while patients with wild-type *KRAS/NRAS/PIK3CA/BRAF* are mostly sensitive to this therapy. Activated tumor driver proteins are shown as frames with thick lines. Refer to the text for details.

In normal cells, upon growth factor (EGF) stimulation and the activation of its receptor (EGFR), the inactive RAS protein (GDP-bound RAS) changes transiently to the active form (GTP-bound RAS) (Figure 5). However, KRAS, which is frequently mutated in mCRC, sustains the activated RAS-GTP form. This causes constitutive activation of downstream effectors, mainly RAF in the RAF-MEK-ERK pathway, PIK3CA (p110α) in the PI3K-AKT-mTOR pathway, and RALGDS in the RALGDS pathway, as reviewed previously [35]. The activated RAS-GTP form takes on an “open” conformation in the Switch I and Switch II regions (amino acids 28-63), allowing the effector domain of residues 32 to 40 within Switch I to interact with the Ras binding domain (RBD) of RAF, while PIK3CA (p110α), and RALGDS bind at both the Switch I and Switch II regions [85]. The mechanisms involved in signaling to downstream effectors and membrane anchoring of Ras isoforms were reviewed by Castellano and Santos [99].

Inhibition of overexpressed EGFR has been used in cancer therapies [42]. For mCRC, it was reported that patients with amplified EGFR copies respond well to anti-EGFR treatment [100]. However, starting in 2006, anti-EGFR therapy with a monoclonal anti-body (mAb) or tyrosine kinase inhibitor (TKI) was reported to be less effective in mCRC patients with *KRAS* mutations at codon 12/13 [101, 102] because the mutated *KRAS* oncogene is unaffected by EGFR signaling (Figure 5). Additional studies have shown that patients with *NRAS, BRAF,* and *PIK3CA* oncogene mutations (shown by circles with thick lines) are also resistant to anti-EGFR therapy [103]. The proportions of patients with each of these activated oncogenes among anti-EGFR-resistant patients and patients with large intestine cancer (COSMIC) were similar - i.e., highest for *KRAS,* followed by *PIK3CA, BRAF,* and *NRAS* [103]. Thus, activated *KRAS* mutations were the first to be reported in anti-EGFR-resistant mCRC patients. Some patients had both *PIK3CA* and *KRAS* mutations [103]. This finding was consistent with the presence of another direct signaling pathway from EGFR to PI3K [104]. PTEN mutations also contribute to anti-EGFR treatment resistance [105], since, like PIK3CA mutations, they enhance AKT phosphorylation [106].

All activated oncogenes detected through anti-EGFR therapy was found to be a transformation-driver *in vitro* using cultured cells [35, 107–109]. Further, with the exception of *NRAS*, they were also the first to be identified in retroviruses [36]. These mutated oncogenes were thus acquired/selected during anti-EGFR therapy, and work individually as “oncogenic drivers for tumor initiation and progression and therefore conferred primary/acquired resistance to cells during anti-EGFR therapy. Figure 5 shows that these activated oncogenes transfer malignant signaling to their downstream effectors to promote tumor growth and act as tumor drivers independently of upstream signaling. By contrast, their wild-type forms are regulated by upstream proteins.

Growth factor receptors, including MET, HER2, and HER2/HER3 heterodimer, activate both the RAS pathway and a direct PI3K pathway. Amplification/activation of these receptors or enhanced expression of their ligands, HGF and TGF alpha, also conferred resistance to anti-EGFR therapy [110–113]. Acquired EGFR mutation, which abrogates the binding of the mAb cetuximab has been reported [114]. The mechanisms underlying resistance to anti-EGFR therapies have been reviewed for mCRC [115–117].

As *KRAS* mutations predict the efficacy of anti-EGFR therapy, *KRAS* mutation testing is mandatory, and anti-EGFR therapy is only prescribed for mCRC patients with wild-type *KRAS* (see Figure 1). Consequently, molecular epidemiological data of *Kras* mutations in mCRC patients has accumulated. *KRAS^G12D^* and *KRAS^G12C^* were the most common *KRAS* genotypes in mCRC and lung cancer patients, respectively [118]. Each mutant genotype has been correlated to clinical outcomes [119–121].

#### Activated *KRAS* as a therapeutic target

As *KRAS* was found to be the most frequently mutated oncogene in human adenocarcinomas, the development of drugs targeting activated *KRAS*/KRAS has been pursued. However, initial attempts to block Ras family proteins by lipid modification of HVR were unsuccessful, as described by Ledford [122]. Targeting *KRAS* /KRAS sequences in genomic DNA, mRNAs, and proteins, in addition to cancer cells with mutated *KRAS*, has been explored as described below.

1. Targeting oncogenic codon 12 sequences of chromosomal *KRAS* DNA.
  This was accomplished by alkylating adenine N3 at the target sequence. The modification induced DNA strand cleavage and thereby suppressed the growth of *KRAS*-mutated human colon cancer cells [123]. This approach was suitable for inactivating either *G12D* or *G12V* mutated genes, which are frequent in mCRC, [118].
2. Silencing of *KRAS* mRNAs using siRNA [124, 125] or miRNAs (such as Let-7 [126] or miR143 [127]), which indirectly suppress levels of mutated KRAS proteins in cancer cells. A new administration method was developed to maintain siRNA stability and to introduce siRNA slowly into *KRAS G12D*-mutated pancreatic carcinoma tissue [125].
3. KRAS^G12C^ protein was modified by guanine nucleotide (GN) analogs so that the analogs formed a covalent bond to the cysteine residue at codon 12 in the GTP/GDP pocket [128]. The modified KRAS protein is unable to bind to downstream effector proteins, and oncogenic signaling is thereby attenuated, as reviewed by Wang et al. [129]. Most *KRAS^G12C^* mutations are due to G to T nucleotide transversion in lung cancer [2].
4. Therapeutic immunization (GI-4000 vaccine) with whole cell lysates of recombinant yeast that expresses a mutated KRAS protein has been reported [130, 131].
5. Eradication of *KRAS*-activated cancer cells *in vivo* by T-cell response. Anti-CTLA-4, anti-PD-1, or anti-PD-L1 antibodies generated T-cell responses against cancer-specific neo-antigens. This method was effective in patients suffering from malignant melanoma with mutated *NRAS* or *BRAF* [132]. The possible use of these types of antibodies was reported for *KRAS-* mutated lung cancer patients, since higher PD-1 or PD-L1 levels were observed in these patients [133, 134].

Two splice variants of *KRAS* mRNAs differing mainly in HVR are produced from the *KRAS* oncogene by alternative splicing: *KRAS^4A^* and *KRAS^4B^*. Relative amounts of the mRNA species were approximately equal in most samples from 17 fresh human colorectal tumors, although the *KRAS^4B^* species is more abundantly expressed in cell lines [135].

Activated *KRAS^4B^* had been reported to stimulate cell migration more strongly than *KRAS^4A^*, while the opposite was true for focus formation and anchorage-independent growth. Cell motility facilitates angiogenesis, invasion, and ultimately metastasis. It had been suggested that both splice variants might act together to confer the fully malignant phenotype induced by activated *KRAS* [136]. KRas^4A^ shares lipid modifications of HVR as well as a G-domain sequence with NRAS and Hras, while the sequence and lipid modification of KRas^4B^ HVR are distinct from the above 3 RAS proteins (see Figure 3C). Therapy inhibiting both KRAS splice variants or their downstream effectors, such as the combination of MEK and PI3K inhibitors may prove an effective strategy for the treatment of *KRAS*-mutated cancers [137–139]. Therapeutic strategies against KRAS were recently reviewed by McCormick [140].

In conclusion, the discovery of Kirsten *Ras* oncogene sequences in both viral genomes and transforming DNA fragments in cancer cells lead to the discovery of crucial roles for *KRAS* in human carcinogenesis, as well as the high frequency of *KRAS* mutations in human cancer. Furthermore, recent results regarding anti-EGFR therapeutic efficacy in patients bearing wild-type or mutant *KRAS* suggested that it is the most important cancer driver *in vivo*. Improved knowledge of the complex mechanisms involved in *KRAS* activation and signaling is necessary in order to develop new therapeutic strategies directed against mutated *KRAS*.
